# Retraction Note to: The protective effect of Pentoxifylline on testopathy in male rats following Dimethyl Nitrosamine administration: An experimental study

**DOI:** 10.18502/ijrm.v17i12.6550

**Published:** 2020-02-27

**Authors:** Mohammad Reza Salahshoor, Azita Faramarzi, Shiva Roshankhah, Cyrus Jalili

Retraction to: Int J Reprod BioMed (2019) 17: 727-738

doi.org/10.18502/ijrm.v17i10.5292

This article has been retracted at the request of the IJRM editorial board, based on the results of an investigation which found serious flaws in the study results.

The online version of the original article can be found at http://journals.ssu.ac.ir/ijrmnew/article-1-1695-en.html.

Salahshoor MR1, Faramarzi A1,2, Roshankhah Sh1, Jalili C2.

1Department of Anatomical Sciences, Medical School, Kermanshah University of Medical Sciences, Kermanshah, Iran.

2Fertility and Infertility Research Center, Health Technology Institute, Kermanshah University of Medical Sciences, Kermanshah, Iran.

Corresponding Author:

Cyrus Jalili: Department of Anatomical Sciences, Daneshgah Ave., Taghbostan, Kermanshah, Iran. Postal Code: 6714869914

Salahshoor MR

Email: reza.salahshoor@yahoo.com

Faramarzi A

Email: a.faramarzi90@gmail.com

Roshankhah Sh

Email: roshankhah@yahoo.com

Jalili C

Email: cyja2019@gmail.com

